# SeqRate: sequence-based protein folding type classification and rates prediction

**DOI:** 10.1186/1471-2105-11-S3-S1

**Published:** 2010-04-29

**Authors:** Guan Ning Lin, Zheng Wang, Dong Xu, Jianlin Cheng

**Affiliations:** 1 Informatics Institute, University of Missouri, Columbia, Missouri, 65211, USA; 2 Department of Computer Science, University of Missouri, Columbia, Missouri, 65211, USA

## Abstract

**Background:**

Protein folding rate is an important property of a protein. Predicting protein folding rate is useful for understanding protein folding process and guiding protein design. Most previous methods of predicting protein folding rate require the tertiary structure of a protein as an input. And most methods do not distinguish the different kinetic nature (two-state folding or multi-state folding) of the proteins. Here we developed a method, SeqRate, to predict both protein folding kinetic type (two-state versus multi-state) and real-value folding rate using sequence length, amino acid composition, contact order, contact number, and secondary structure information predicted from only protein sequence with support vector machines.

**Results:**

We systematically studied the contributions of individual features to folding rate prediction. On a standard benchmark dataset, the accuracy of folding kinetic type classification is 80%. The Pearson correlation coefficient and the mean absolute difference between predicted and experimental folding rates (sec^-1^) in the base-10 logarithmic scale are 0.81 and 0.79 for two-state protein folders, and 0.80 and 0.68 for three-state protein folders. SeqRate is the first sequence-based method for protein folding type classification and its accuracy of fold rate prediction is improved over previous sequence-based methods. Its performance can be further enhanced with additional information, such as structure-based geometric contacts, as inputs.

**Conclusions:**

Both the web server and software of predicting folding rate are publicly available at  http://casp.rnet.missouri.edu/fold_rate/index.html.

## Background

Protein folding is one of the most important problems in molecular biology. Two main aspects of the folding process concern the kinetic order and the rate constant. The kinetic order of the protein folding indicates whether the sequence reaches its native structure through intermediate states or not. The folding rate is inversely proportional to the time that the protein needs to collapse into its stable tertiary structure. Proteins have very different rates of folding. Some of them fold within microseconds [1]; some need an hour to fold [2]. Small proteins often (but far from always) fold faster than the larger ones [3]. Many studies have been conducted to estimate protein folding rates based on either experimental protein structural information [4-7] or protein homology sequence searches using databases [8]. However, since only limited amount of experimental folding rates is available for database search and most proteins do not have solved experimental structures, prediction of folding rates based on sequence only has been a logical choice for researchers lately.

Various theories and simulations suggest a surprising simple linear relation between the number of residues in a protein, its length L, and the rate at which it folds. It is in the form of

where *k_f_* is the experimental folding rate, L is the length of the protein, and C1 and C2 are simple constants [3,9-12]. The correlation between folding rates and protein sizes is stronger for multi-state proteins that have folding intermediates, and weaker for two-state proteins that do not have such intermediates [3]. The above formula is a good estimate for the multi-state folding proteins, but not for single-domain two-state folders. In other words, protein length does not describe the transition rates of direct folding well.

In 1998 Baker and co-workers [13] found a strong correlation between the native topological complexity, defined by the parameter contact order (CO), which uses the information about the average sequence separation of all contacting residues in the native state of two-state proteins, and the folding rates of 12 two-state proteins. The correlation between protein-folding rates and their hierarchical structures (secondary structure and structural topology) suggests that hierarchical information could be one of the key features for determining folding rate. Although folding rates of proteins of both two kinetic pathways (i.e. two-state and multi-state folding) can be roughly predicted from the protein secondary structures [14], the prediction scheme should be adjusted to accommodate the differentiation of the two kinetic pathways to improve the accuracy [15].

In the past years, various approaches have been designed to estimate the logarithm of the two-state folding rate starting from using structural information. Several methods based on correlation between the logarithm of the folding rate and structural predictors such as Contact Order (CO) [13], Long-range Contact Order (LRCO) [16] (contact between two residues that are close in space and far in the sequence), total contact distance [17], effective length of folding chain [14] or Geometric Contact (GC) [18] have been developed. These methods require the tertiary structure of a protein as input to predict its folding rate. Since the vast majority of proteins’ tertiary structures are still not solved, it is important to design methods that can predict folding rate from protein sequence directly. Toward this goal, in the seminal work [19], Punta and Rost first showed LRCO had better correlation with folding rates than CO. Then they used LRO values predicted from protein sequences for folding rate predictions and achieved 0.61 correlation between the predicted and true folding rates for a set of two-state folding proteins.

Most of folding rate prediction methods are knowledge-based approaches that build a function to map input predictors (e.g. contact order) to folding rates. Traditionally these methods used only a single estimator, either CO, LRCO, or chain length to design linear models between these predictors and protein folding rates. Recently Huang et al. showed that the linear combination of several predictors, such as amino acid rigidity (R), composition vectors (CV), chain length (L), amino acid weight (W), degeneracy (D), and composition index (CI) can increase the correlation between predicted and actual two-state folding rates [15], although the relationship between some of these predictors and the folding rate may not be linear.

Besides folding rate prediction, some studies also have been done to classify the proteins into different folding classes based on their secondary structures. Some classified folders into all- *α *-class, all- *β* -class and *α* / *β* -class [20,21], and some even classified folders into 83 different classes [22]. Interestingly, not much has been done to classify the proteins folders based on their binary folding kinetic mechanisms, such as two-state folders or multi-state folders.

A few applications and web servers have been developed for protein rate predictions, such as FOLD-RATE [23], and PPT-DB [8], but not yet for fold kinetic classification. In 2007, K-Fold has been developed as the only folding kinetic classification tool so far, but it trained the classification using experimental 3D structural information instead of just using sequence information and it also used same rate prediction models for both two-state and multi-state proteins [7]. FOLD-RATE predicts folding rate using amino acid sequence and 49 amino acid properties. It separated proteins into all-α, all-β and mixed class first, then used multiple regressions for folding rate prediction, while PPT-DB is a database which uses homology sequence search.

Here we developed a non-linear machine learning method (Support Vector Machine classification and regression) that can not only classify proteins into two-state or multi-state folders, but also predict folding rates, using only the information extracted from the amino acid sequence of a protein, without any explicit knowledge of the experimental tertiary or secondary structures. We used a large set of features including protein sequence length, predicted LRCO, predicted long-range contact number (LRCN), predicted *α*-helical content and *β* -sheet content and amino acid composition with non-linear SVM models for both protein binary kinetic classification and folding rates prediction. Some features such as secondary structure composition and amino acid composition are new. And our method of deriving LRCO and LRCN are based on predicted residue-residue contact probabilities instead of binary contacts used by previous work [19]. We used both Pearson correlation and MAD (mean absolute difference) as accuracy measurements between predicted rates and actual experimental rates. Our method performs favorably when compared to other sequence-based methods. We also developed a web server with name ‘SeqRate’ for the method at our site: http://casp.rnet.missouri.edu/fold_rate/index.html.

## Results and discussion

### Predicted contact vs. Real contacts

We compared the LRCOs and LRCNs estimated from sequence and calculated from structural information obtained from PDB [34] to see how well they correlate with folding rates. Table 1 shows the correlations between two-state protein folder folding rates and each estimated and real contact predictor using both two-state and multi-state protein folders from IvankovData. The correlations between estimated contacts and real contacts are above 0.7. And the estimated contacts predicted from sequences have correlation to folding rates only about 3 ~ 5% worse than real contacts in both two-state folders and multi-state folders. Therefore, estimated contacts can be used in place of real contacts without losing too much information. From this onward, if not mentioned specifically, ‘LRCO’ and ‘LRCN’ will indicate estimated LRCO and LRCN from sequences. The negative correlation between LRCO (resp. LRCN) and folding rate on two-state folders is stronger than multi-state folders.

**Table 1:** Correlation between estimated and real contacts and experimental folding rates

	estLRCN	rLRCN	estLRCO	rLRCO	rate
estLRCN	1 (1)	**0.78 (0.75)**	0.95 (0.84)	0.61 (0.54)	**-0.68 (-0.55)**
rLRCN	-	1 (1)	0.79 (0.75)	0.87 (0.81)	**-0.71 (-0.58)**
estLRCO	-	-	1 (1)	**0.82 (0.74)**	**-0.72 (-0.48)**
rLRCO	-	-	-	1 (1)	**-0.77 (-0.51)**
rate	-	-	-	-	1 (1)

Correlation between folding rates and estimated long-range contact number (estLRCN), estimated long-range contact order (estLRCO), real long-range contact number (rLRCN) and real long range contact order (rLRCO) using 37 two-state proteins in IvankovData and 24 multi-stat proteins in IvankovData (data shown in the parentheses).

### Effectiveness of each feature in folding rate prediction

In order to test the effectiveness of each individual feature, we used each feature as input to predict folding rate separately through SVM regression. Two different measures were applied to evaluate the performance of the results. One is Pearson correlation coefficient between predicted rates and experimental rates. The other measure is mean absolute difference (MAD), which measures how much predicted values deviate from real values. The correlation coefficient and MAD were calculated for two-state and multi-state proteins separately. Each feature-specific SVM prediction model was trained using leave-one-out procedure and used to predict the folding rate on the left-out protein. Table 2 demonstrates the general trend of understanding, which is protein sequence length has more than two times higher correlation values with multi-state folders than two-state folders, and protein topologies (e.g. secondary structure information) have almost twice correlation values with two-state folders as with multi-state folders. These strongly kinetically biased features support the need of separate prediction models for different folding kinetic.

**Table 2:** Correlation between predicted folding rates and experimental folding rates using sequence length and other estimated predictors on IvankovData.

	L	LRCO	CO	LRCN	*α*-helical content	*β* -sheet content	Coil content
Two-state folding rate	-0.32	0.72	0.61	0.68	-0.51	0.57	0.13
Multi-state folding rate	-0.80	0.46	0.33	0.55	-0.18	0.11	0.05

L = protein sequence length, LRCO = estimated long-range contact order, CO = estimated contact order in [15], LRCN = estimated long-range contact number. IvankovData is used and there are 37 two-state proteins and 24 multi-state proteins.

LRCO yields the best performance with correlation 0.72 for two-state proteins while protein sequence length demonstrates the best negative correlation of 0.8 for multi-state proteins. For both two-state and multi-state folders, LRCO was preferred over CO since it has higher correlations in both folding kinetics. On multi-state proteins contact number performs the second best with correlation 0.55. Note that the correlation using estimated LRCO on two-state proteins is 0.72, higher than CO has, which is 0.61 reported in [19] on the same data set, indicating that LRCO calculated from contact probability map in our method might be more informative than that derived from binary contact map used in [19].

Coil content has low correlations, 0.13 and 0.05, with both two-state folders and multi-state folders respectively; therefore it is not used in building either folding rate prediction model. Also *α* -helical content and *β* -sheet content have low correlation values of -0.18 and 0.11, respectively in multi-state folders, therefore both features are not included for the multi-state folding rate prediction model. Actually by including *α *-helical content and *β* -sheet content as features, the prediction results have shown no changes, neither increasing nor decreasing accuracies.

One feature needed to be mentioned here and is not shown on Table 2 is amino acid composition, which is a set of 20 amino acid frequency values. It has shown to be a relevant feature for deciding folding kinetic [29,30]. It was included as one of our classification features, but it has shown weak correlations with folding rates of both folding kinetic orders in our results. Our tests have indicated the overall correlations of amino acid compositions with the folding rates are only around 0.3. Therefore, this feature is not used for SVM regression rate prediction model in order to avoid over-fitting.

### Sequence-based folding kinetic type classification

**Figure 1:**
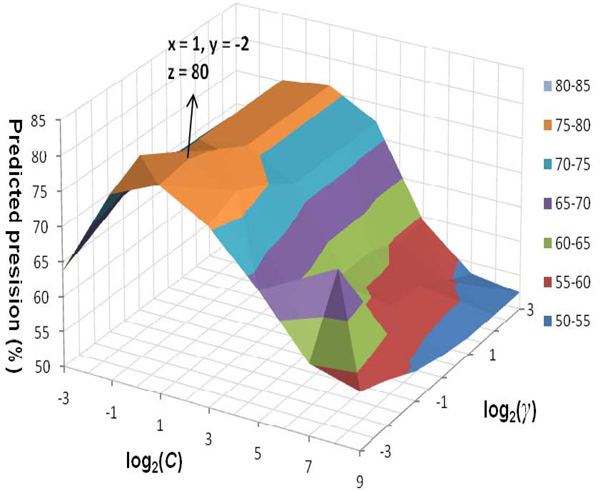
Classification accuracy surface vs. variations of parameters C and γ.

Protein sequence length and protein topologies are both favorable folding rate determination factors for two folding types. Protein sequence length is a good predictor in multi-state folder rate prediction, but not in two-state folders. And protein topologies have better correlations with two-state folding rates than multi-state folding rates. We built an SVM classification model based on sequence length, estimated LRCO and CN, *α *-helical content, *β* -sheet content and 20 frequency values of amino acid compositions. As in other multivariate statistical models, the performances of the SVM for classification depend on the combination of several parameters. In general, the SVM classification involves two classes of parameters: the parameter C for the trade-off between training error and margin size and kernel function parameters such as inverse of variance γ for Gaussian kernel. To maximize the performance, we performed the parameter optimization using a grid search approach within a limited range. The classification model is trained and tested using leave-one-out cross-validation (LOOCV). Figure 1 shows the profile of classification accuracy vs. the variations of parameters C and γ. The prediction accuracy profile peaked at (C, γ) = (1, 0.25). The best classification accuracy of using Gaussian kernel function is 80%, to our best knowledge, which is higher than any of other classifiers in the literature.

We have used other kernel functions, namely linear, sigmoid and polynomial functions on SVM model for the same data set. The accuracies of those three kernels were 62%, 50% and 72%, respectively, lower than that of using the Gaussian kernel. Comparisons of different features impacts on classifications have also been performed. Interestingly, sequence length has dominant impact on classification result. By including sequence length, prediction accuracy is improved by 35%.

### Linear regression model for fold rate prediction using sequence-based estimated multi-predictors

To test if multiple features contain complementary information, we started the analysis by a linear combination of multiple features. We use the linear regression analysis to build a model for folding rate prediction. R package [35] has been used for ANOVA analysis to obtain the RMSE (Root mean square error), F-value and P-value for each regression test. Four regression tests have been devised as shown in Table 3. The table shows the linear regression using all four features yields the best correlation, 0.72, between predicted rates and real rates out of different selections of predictors, with P-value of 7.3e-06. The result confirms that using multiple features for protein folding rate prediction performs better than single predictor.

**Table 3:** Linear regression analysis using different combinations of predictors

	estCN	estLRO	estLRO+estCN	*α* + *β* Contents	All Predictors
Cor2Rate	0.64	0.66	0.69	0.67	0.72
RMSE	1.3	1.14	1.12	1.27	1.16
F-value	21.27	37.03	38.69	34.66	35.52
P-value	6.9e-05	1.1e-06	8.7e-07	6.5e-05	7.3e-06

Results of linear regression analysis using R package [35] with different combinations of predictors, such as etsCN (estimated Contact number), estLRO (estimated Long range order), estLRO+estCN (using combination of predictors estCN and estLRO), *α* + *β* Contents (using combination of predictors *α*-helix content and *β* -sheet Content) and All Predictors (using all 4 predictors). Results can be shown as Cor2Rate (correlation between estimated rates and real rates using selected predictors), RMSE (Root mean square error for the regression), F-value and P-value.

### Sequence-based fold rate prediction using multiple features and non-linear SVM regression

We selected four predictors including LRCO, CN, *α *-helical content, and *β* -sheet content with SVM to predict two-state folding rates. Besides two parameters C and γ used for SVM classification, SVM regression requires additional important parameter ε (regulate regression tube width) for performance optimization. Due to the intensive computational nature of grid search algorithm in high dimensions, we performed the tuning of parameter set (C, γ, ε) heuristically. We first obtained the optimal parameter values for C and γ with the fixed value of ε = 0.1 (default SVM value), then searched for the best value for ε. With the same procedure we did for SVM classification, we obtained the optimal parameter set of (C, γ, ε) = (8, 0.125, 0.1) for constructing prediction model.

Five different sets of training and testing data were generated. Each one was generated by randomly selecting 10% for testing and rest 90% for training from IvankovData. Then five different SVM prediction models using optimal parameter set was trained using leave-one-out cross-validation (LOOCV) and predicted on the test data sets. The average correlation and MAD are 0.81 and 0.78, respectively, from five test sets. The results are substantially better than the linear combination of multiple features, indicating the relationship between the features and folding rates is probably non-linear

For multi-state folder rate prediction, one extra predictor, protein sequence length, besides four other predictors used for two-state folders, was included for the SVM regression to predict multi-state folder’s rate.

Our multi-feature SVM-regression method is comparable with or better than other sequence-based methods in Table 4. Our method not only has better correlation between predicted rates and experimental rates than all the sequence-based method except FOLD-RATE, but also has smaller MAD values between predicted and real rates than all the sequence-based methods. FOLD-RATE has obtained the highest 0.91 correlation between predicted and experimental rates, but its mean absolute difference between predicted and experimental values is around 1.1, which is higher than our method. The reason could be due to FOLD-RATE breaks proteins into structural classes for individual training, which largely decrease the number of proteins per structural class, resulting in high correlation but high variance between predicted and real values. K-Fold uses experimental protein kinetic and structural information to estimate folding rates and has achieved 0.81 classification accuracy for folding types, but has correlation value of 0.74 between predicted rates and experimental rates, lower than our method using sequence information only. Our sequence-based method has the kinetic type classification accuracy of 0.80, which is very close to that of K-fold.

**Table 4:** Comparison among different folding rate prediction methods based on “IVANKOVDATA”

Methods	Method Type	Fold kinetic Classification Accuracy	Correlation	MAD
Effective length method	sequence	NA	0.70	0.96
LRCO method	sequence	NA	0.61	0.81
FOLD-RATE	sequence	NA	0.91	1.1
K-Fold	structure	81%	0.74	0.75
Multi-predictor SVM (two-state)	sequence	80%	0.81	0.79
Multi-predictor SVM (multi-state)	sequence	80%	0.80	0.68

Method 1: Effective length method [14]

Method 2: LRCO method [19]

Method 3: FOLD-RATE [16]

Method 4: K-Fold [7]

Method 5: Our multi-predictor SVM (two-state)

Method 6: Our multi-predictor SVM (multi-state)

Method-Type means if the method is using experimental structural data (structure) or using only sequence data (sequence). Correlation here means the correlation value between predicted rates and experimental rates. MAD is mean absolute difference between predicted rates and experimental rates.

To study how the classification model and two separate fold kinetic models would affect the results, we investigated a few cases. Chromosomal protein Ubiquitin (PDB ID: 1UBQ) has a sequence length of 76 amino acids and experimental folding rate of 7.3 (in natural-base logarithm scale) in the unit of sec-1. It has been used by many researchers as multi-state folder [14,18,19,36], but later it was shown experimentally to be a two-state folder [8,37]. Assuming 1UBQ as multi-state folder, we used the multi-state prediction model and obtained fold rate of 3.97. But after being correctly classified into two-state using our SVM classification model, a value of 6.21 was obtained, which is much close to the experimental rate. DNA-binding protein Engrailed Homeodomain (PDB ID: 1ENH) is another example of such a case. It has a sequence length of 16 and folding rate of 10.5 (in natural-base logarithm scale) in the unit of sec-1. Assuming it was as multi-state [38], then the predicted folding rate would be 2.55. However, our classification model has classified 1ENH as a two-state folder and we used two-state prediction model to predict the folding rate as 10.05. 1ENH has been shown and used as two-state folder in later literatures [14,18,19]. These examples demonstrated that our folding type classifier can help correct errors in manual folding type classifications.

### Results of using geometric contacts derived from tertiary structures

To test if structural information can improve our method, we added another feature, geometric contact (GC) derived from experimental tertiary structures [18], to predict folding rates. The GC number, *N_α_*, which is the number of well-packed nonlocal contacts, was shown to have significant correlations of -0.86, -0.86 and -0.83 for two-state proteins, multi-state proteins and all proteins combined, respectively [18]. Using a 20-dimensinal vector recording the number counts of the 20 residue types in geometric contact for rate prediction, correlation of 0.82 and MAD of 1.34 between predicted rates and experimental rates were achieved by using linear regression for all proteins combined [18].

To test the impact of geometric contacts on our method, we used the geometric contact as additional feature to predict folding rates. We were able to obtain the singular values of geometric contacts, instead of 20-dimensinal vector, from Zhang and Liang for the ZhangData set. Therefore, we performed the SVM-regression prediction on two-state folders and multi-state folders separately on the ZhangData set, and then all proteins together. The correlation of 0.87 can be achieved for 45 two-state folders with smallest MAD value of 0.78, correlation 0.85 for multi-states with MAD 0.72, and correlation 0.85 for all protein folders with MAD 0.91, The improvement over the -the results obtained by Zhang mentioned above is probably due to two factors: robustness of non-linear SVM regression and additional sequence-based predictors.

## Conclusions

We have developed a new protein fold rate prediction method (SeqRate) using Support Vector Machine regression with a set of features derived from protein sequences only. As the first method that can predict protein folding kinetic types from protein sequences, it achieved the accuracy comparable to the methods based on experimental structures. The accuracy of fold rate prediction of the method was also improved over previous sequence-based prediction methods. Its performance can be further improved with the addition of structure-based features. SeqRate is a fast and robust method suitable for large-scale protein folding rate prediction. The web server of SeqRate for protein folding rate prediction is available at http://casp.rnet.missouri.edu/fold_rate/index.html.

## Methods

### Data sets

We used two data sets of proteins with experimentally determined folding rates. Both data sets contain two-state folders and multi-state folders. One data set contains 24 multi-states folders and 37 two-state folders, and is referred to as “IvankovData” composed by Ivankov in 2004 and also used in [19]. This data set is used to train and test support vector machines (SVM) to predict both folding type and folding rate. The folding rate is in the unit of sec^-1^ and transformed in the base-10 logarithmic scale. The other data set [18] contains 34 multi-state folders and 45 two-state folders, and is referred to as “ZhengData”. This data set is mainly used to test the improvement of adapting extra predictor, such as ‘geometric contact’, to the current prediction model. Structural information of protein is obtained from the Protein Data Bank (PDB) [24].

### Methodology

Our method for protein folding rates was developed based on an SVM. In this study we divide our protein rate prediction into two steps. First we use SVM classifier to classify folding types based on binary kinetic mechanism (two-state or multi-state), instead of using structural classes of all- *α* -class, all- *β* -class and *α* / *β* -class. The second step of protein rate prediction is developing two separate SVM regression prediction models for two-state folders and multi-state folders, considering different folding behaviors between these two types. In this study, multiple input features derived from protein sequences were used in protein folding type classification and folding rate prediction. We also studied the impacts of using different input features, such as protein chain length and several protein topology features [25], on folding kinetic classification and rate prediction for two-state and multi-state folders.

### Input features

Features, such as protein sequence length, long-range contact order, long-range contact number, *α *-helical content, *β* -sheet content and amino acid compositions, used in SVM training models, are defined and discussed as follows.

*Protein sequence length.* Protein sequence length is the number of residues in the chain that has been used or would be used for experimental folding rate tests. It has been revealed that chain length is an important factor for determination of protein folding rates [14,26,27], although it is insufficient to just use sequence length to determine the folding type. Smaller sequences usually tend to fold with simpler folding mechanism without any intermediate state like in multi-state proteins.

*Contact order (LRCO) and contact number (LRCN).* LRCOs and LRCNs used in this study were both calculated based on contact map generated from the SCRATCH suite [6] using protein sequences as inputs. A protein contact map, a two-dimensional matrix, represents the distance information between every two residues’ C-alpha atoms of a three-dimensional protein structure. SCRATCH was used to predict the contact probability matrix *P* for the probabilities of any pair of residues contacting with each other, i.e. the likelihood that their distance is below a threshold. The distance threshold used here is 8 Å and the sequence separation is at least 12 amino acids apart. An element *P_ij_* in the matrix is the predicted probability that residues *i* and *j* are in contact. As in Reference [16], only long-range contacts (i.e. sequence separation of |*i*-*j*| ≥ 12) were used to derive contact order and contact number.

The LRCN is defined as the expected number of long-rage contacts in a protein. So far, most methods first derive a binary contact map from a probability contact map according to a probability threshold and then count the numbers of contacts [19]. Here, we introduce a modified method to directly calculate contact number from contact probability map and it is further normalized by the power of sequence length. Then the contact number is defined as following

LRCN= (1)

where *P_ij_* is the contact probability of residue *i* and *j*, which should be no more then 8 Å away and at least 12 sequence separation apart; *L* (sequence length) to the power of *c* is used to normalize contact number. *c* is set to 1 as in [19].

Different from LRCO (Long-range Contact Order) calculation based on binary contacts in [19], we calculated contact order from contact probabilities as following

LRCO = (2)

where *P_ij_* is the probability of residues *i* and *j* within 8 Å when at least 12 sequence separation apart; *L* (sequence length) to the power of *c* is used to normalize contact order. Just as the calculation in LRCN, probabilistic real values of contacts are used in the formula. *c* is set to 2 as in [19].

*Secondary structure composition.* Rose and collaborators [28] observed that folding rates correlate well with the overall secondary structure composition in three states (helix, strand, coil) assigned from 3D coordinates. So we used the predicted percentages of helix, sheet and coil contents of a protein as additional inputs for folding rate prediction. Secondary structures were predicted by SCRATCH [6].

*Amino acid composition.* Amino acid composition has been shown to be relevant to protein folding types and a good indicator for folding type identification [29,30]. The basic assumption is that if certain amino acids are optimal for protein structure, natural selection should have acted over evolutionary time to increase the frequency of these amino acids. Therefore, proteins with different amino acid composition would have different folding rates and folding types. In 2007, Ma and his colleagues demonstrated some of contents of amino acids differed between two-state and multi-state folders in a significant level of *p*<0.01 [31]. Here we use the each amino acid occurrence frequency in the protein sequence as amino acid composition. Then, each of 20 amino acid compositions is used as one input feature for SVM.

### SVM training and learning procedure

A Support Vector Machines (SVM) is an advanced machine learning method, characterized by usage of kernels, absence of local minima, sparseness of the solution and capacity control obtained by acting on the margin or number of support vectors [32]. It has a set of related supervised learning methods and can be applied to both classification and regression problems. In this study, we used a well-implemented SVM toolbox, SVM-light [33] to first build a classification training mode for folding kinetic binary classification, and then construct the two separated SVM regression models to predict protein folding rates for both two-state folders and multi-state folders using multiple predictors mentioned above as inputs. We applied the radial basis Gaussian kernel in our experiments. All training and testing procedures mentioned in this study using SVM models were performed and validated in strict Leave-One-Out Cross-Validation (LOOCV) process.

## Competing interests

The authors declare that they have no competing interests.

## Authors' contributions

GNL carried out the SVM-regression and classification experiments for folding rate analysis and prediction, implemented the software in Java, and drafted the manuscript. ZW constructed the web-server for the SVM folding rate prediction. DX provided some ideas and formulations for experiment design and critically revised the manuscript. JC conceived the study, designed the experiments, directed the project, and critically revised the manuscript. All authors read, edited and approved the final manuscript.
